# The Use of Protective Equipment and Personal Monitoring in Fluoroscopy-Guided Procedures: A Case Study in Portugal

**DOI:** 10.3390/healthcare14091196

**Published:** 2026-04-29

**Authors:** Marina S. Cunha, Sara Videira, Matilde A. Rodrigues, João Martins, Manuela V. da Silva

**Affiliations:** 1 Environmental Health, ESS, Polytechnic of Porto, Rua Dr. António Bernardino de Almeida nº400, 4200-072 Porto, Portugal; marinasofia.sc@gmail.com (M.S.C.); mar@ess.ipp.pt (M.A.R.); 2 Radiotherapy Department, Instituto Português de Oncologia do Porto, Rua Dr. António Bernardino de Almeida, 4200-072 Porto, Portugal; 3 Faculty of Engineering, University of Porto, Rua Dr. Roberto Frias, 4200-465 Porto, Portugal; up202312047@edu.fe.up.pt; 4 Department of Radiology, Unidade Local de Saúde de Santo António (ULSSA), Largo Professor Abel Salazar, 4099-001 Porto, Portugal; 5 REQUIMTE/LAQV, ESS, Polytechnic of Porto, Rua Dr. António Bernardino de Almeida nº400, 4200-072 Porto, Portugal; 6 RISE-Health/TBIO, ESS, Polytechnic of Porto, Rua Dr. António Bernardino de Almeida nº400, 4200-072 Porto, Portugal; 7 ESS, Polytechnic of Porto, Rua Dr. António Bernardino de Almeida nº400, 4200-072 Porto, Portugal; jom@ess.ipp.pt; 8 CEAUL—Centro de Estatística e Aplicações, Faculdade de Ciências, Universidade de Lisboa, 1749-016 Lisboa, Portugal

**Keywords:** interventional procedures, occupational exposure, radiation protection, safety culture, surgical procedures

## Abstract

**Introduction**: The increasing use of fluoroscopy-guided procedures raises concerns about occupational radiation exposure, underscoring the need for effective radiation protection (RP) practices among healthcare professionals. The primary objective was to evaluate compliance with the use of personal protective equipment (PPE) and dosimeters, and to identify factors influencing safety behaviors among exposed workers. **Methods**: A cross-sectional, exploratory quantitative study was conducted at a hospital centre using a self-administered questionnaire in fluoroscopy-guided operating and interventional settings. The questionnaire collected sociodemographic and professional data, information on RP training, compliance with personal and collective protective equipment, and dosimeter use, as well as perceptions of occupational risk. **Results**: The study included 52 workers. Compliance with PPE use varied across professions, with radiographers reporting significantly higher use of lead aprons/skirt-coats and thyroid shields than other professionals (*p* < 0.05). The RP training was significantly associated with compliance with PPE and dosimeter use (odds ratios: 4.2–8.9). Older age groups reported lower compliance with PPE use. Overall, risk perception of radiation-related diseases was low (46.2%), and no statistically significant association was found between risk perception and reported PPE use. Regarding protection practices, 67% reported appropriate use of the apron/skirt-coat, 65% of the thyroid shield, and 62% of the dosimeter. The main barriers to PPE use were discomfort, weight, and inadequate cleaning, while forgetfulness was the most reported reason for not using the dosimeter. **Conclusions**: Inconsistent use of protective equipment and dosimeters may lead to unnecessary exposure and underestimation of occupational radiation doses, whereas RP training is a key determinant of compliance and a strong safety culture.

## 1. Introduction

In recent years, minimally invasive procedures and fluoroscopy-guided surgeries have increased, driven by the advantages of this technique over open surgery. This trend may increase healthcare professionals’ exposure to ionizing radiation, creating new challenges for occupational radiation protection (RP) [1].

In Portugal, the population of healthcare workers exposed to ionizing radiation increased substantially between 2008 and 2014, from 11,827 to approximately 21,900, corresponding to an estimated 85% increase over the six-year period [2]. Exposure to ionizing radiation is associated with stochastic effects (e.g., cancer) and deterministic effects (e.g., cataracts), which may compromise health, quality of life, and occupational safety. This underscores the need to minimize exposure and continuously monitor doses [3,4]. Radiation protection aims to ensure adequate levels of protection by preventing tissue reactions (deterministic effects) and reducing the probability of stochastic effects to the extent reasonably achievable, without unduly limiting the clinical benefits of procedures involving ionizing radiation [3,5].

Fluoroscopy-guided procedures are characterized by a non-uniform distribution of radiation within the room, exposing all team members. During surgical and interventional procedures, professionals remain close to the radiation source, underscoring the importance of adequate protective measures to minimize occupational exposure while ensuring patient safety [3].

The personal protective equipment (PPE), such as aprons or skirt/vest combinations, thyroid shields, lead goggles, and lead gloves, as well as collective protective equipment, including mobile, suspended shielding, table skirts, and protective drapes, constitute essential barriers against exposure [4]. Individual monitoring using dosimeters allows the estimation of received doses and verification of compliance with occupational dose limits [4,6]. In 2011, the International Commission on Radiological Protection reduced the occupational lens dose limit to 20 mSv/year (5-year average; max. 50 mSv/year), a 7.5-fold decrease from 150 mSv/year [3]. A strong RP culture positively influences safety behavior, underscoring the importance of assessing these practices using validated instruments [7].

Although RP practices have been widely studied, comparative data across professional groups remain limited, particularly regarding the influence of training, age, PPE characteristics, and risk perception on self-reported protective behaviors during fluoroscopy-guided procedures. This gap is especially evident in Portuguese hospital settings. Therefore, this study aimed to characterize self-reported compliance with PPE and dosimeter use among professionals exposed to ionizing radiation during fluoroscopy-guided procedures and to examine their associations with sociodemographic, professional, training-related, and risk-perception factors.

## 2. Materials and Methods

This study employed a cross-sectional exploratory design with a quantitative approach, using a self-administered questionnaire applied in a public hospital in the northern region of Portugal.

Before data collection, an internal assessment was conducted to identify the departments and professionals potentially exposed to ionizing radiation from fluoroscopy equipment used in surgical and/or interventional contexts. The following departments/medical specialties were included: radiology, interventional radiology, operating theatre, ambulatory surgery, gastroenterology, neurosurgery, anaesthesiology, urology, and pediatrics. The target population comprised workers classified as Category A and Category B radiation workers.

The FluoroWorkSafety questionnaire was developed to evaluate RP performance among doctors, nurses, and radiographers participating in fluoroscopy-guided procedures [8]. However, in this study, we will only analyze the closed-ended questions on demographic data, professional activity and training, the use of personal and collective protective equipment, individual monitoring practices, and the presence of occupational diseases. Before the final application, a pre-test was conducted with 11 workers representative of the target population to ensure the clarity, relevance, and comprehensibility of the items [9,10]. This manuscript is based in part on the author’s defended master’s thesis and includes additional analyses of the results [11].

The questionnaire was distributed via Microsoft Forms to 191 eligible professionals, with individual access links sent to their institutional email addresses. The form remained open for one month (4 July–4 August 2024), during which three reminders were issued.

### Statistical Analysis

Data were analyzed using Microsoft Excel (version 2021, Microsoft Corp., Redmond, WA, USA) and IBM SPSS Statistics (version 29, IBM Corp., Armonk, NY, USA). Categorical variables were summarized as absolute frequencies and percentages, and ordinal variables were summarized as medians and interquartile ranges (IQR).

Selected ordinal variables were dichotomized for inferential analyses. Associations between categorical variables were assessed using Pearson’s chi-square or Fisher’s exact test, as appropriate. Odds ratios (OR) with 95% confidence intervals (CI) were calculated for 2 × 2 contingency tables. Multivariable binary logistic regression was used to estimate adjusted odds ratios (aOR) [12], including age group and sex as covariates. Group comparisons for ordinal variables were conducted using the Kruskal–Wallis test with Dunn’s post hoc test and Bonferroni correction. All tests were two-tailed, with *p* < 0.05 considered statistically significant.

## 3. Results

### 3.1. Sociodemographic Characterization of the Sample, Professional Activity, and Training

A total of 52 workers participated (response rate: 27.2%). Most were female (80.8%), aged 40–59 years, and held a bachelor’s degree (69.2%). By profession, 59.6% were nurses, 25.0% radiographers, and 15.4% physicians. Regarding the exposure context, 44.2% were exposed to fluoroscopy in the operating theatre, 30.8% during interventional procedures, 15.4% in both settings, and 9.6% in gastroenterology. Most participants had 2–10 years of exposure (34.6%) and worked for a single employer (94.2%). Weekly exposure was predominantly ≤ 6 h (53.8%), followed by 7–12 h (28.8%).

Half of the participants reported RP training; of these, 23.1% had attended 2–5 courses, with most completing their last training four years prior (Table 1).

The primary source of RP training was the public employer (73.1%; n = 19), followed by personal initiatives, such as webinars, workshops, or conferences (42.3%; n = 11). By profession, 84.6% of radiographers (n = 11) reported receiving RP training, compared with 38.7% of nurses (n = 12) and 37.5% of physicians (n = 3) (Appendix A).

### 3.2. Availability of Protective Equipment

The most frequently available PPE were the radioprotective skirt-coat (92.3%) and the thyroid shield (98.1%), whereas leaded glasses (61.5%) and radioprotective gloves (34.6%) were less commonly provided. Among collective protective equipment, the table skirts and suspended shields were the most frequently reported (46.2% and 28.8%, respectively). Nearly all workers did not have PPE for exclusive use (98.1%), with only one participant reporting a personal apron/skirt-coat provided by the public employer (Appendix B).

### 3.3. Compliance with Protective Equipment Use

**Figure 1:**
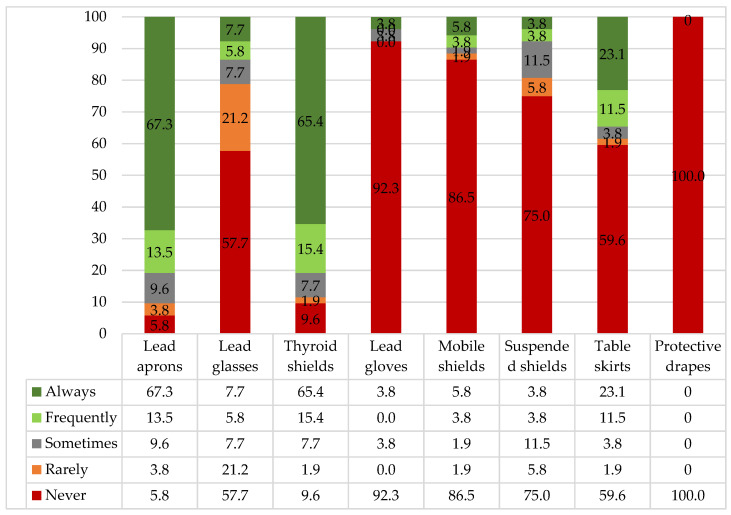
Frequency of protective equipment use (%) among workers (n = 52).

Consistent use (“always”) was highest for the lead apron/skirt-coat (67.3%, n = 35) and the thyroid shield (65.4%, n = 34), whereas substantially lower use was observed for the remaining protective devices (Figure 1).

The main reasons reported for not consistently using the apron or skirt-coat were weight (13.8%; n = 11), discomfort (8.8%; n = 7), and the perception of being sufficiently distant from the radiation source (8.8%; n = 7). Among the two garment types, most workers preferred the skirt/coat (69.2%; n = 36). Notably, 23.1% of participants (n = 12) did not have the option to choose between the two in their workplace.

Among those who did not consistently use the thyroid shield, the most frequently cited reasons were dirtiness (8.3%; n = 5), inadequate disinfection (6.7%; n = 4), and improper storage (6.7%; n = 4). Low compliance with leaded glasses was mainly attributed to their absence (21.6%; n = 16) or unavailability (10.8%; n = 8), while for leaded gloves, the reasons reported were absence (37.1%; n = 23), the perception of being sufficiently distant (14.5%; n = 9), and loss of tactile sensitivity (9.7%; n = 6). The absence of mobile, ceiling, and table shields was the predominant reason for non-use (67.9% (n = 36), 50% (n = 27), and 43.6% (n = 24), respectively). Although available in the institution, the protective drape was not used by any professional, with the most frequently mentioned reasons being its absence (73.6%; n = 39) and the perception that it does not apply to the work area (18.9%; n = 10) (Appendix C).

### 3.4. Compliance with the Use of Individual Monitoring Devices

**Figure 2:**
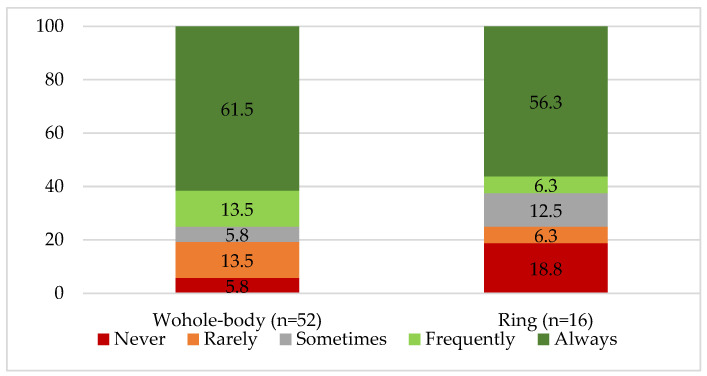
Frequency distribution of whole-body and ring dosimeter use (%) among workers (n = 52).

All participants (n = 52) reported receiving one whole-body dosimeter, and 0% reported receiving an eye lens dosimeter, a wrist dosimeter, an electronic dosimeter, or two dosimeters simultaneously. Of these, 30.8% (n = 16) used both whole-body and ring dosimeters. Adequate use of dosimeters was reported by 61.5% for whole-body dosimeters and 56,3% for ring dosimeters (Figure 2).

Of those using only a whole-body dosimeter, 82.7% placed it at chest level under the apron. Access to dosimeter reports was reported by 76% of respondents; among these, assessments were mainly conducted quarterly (46.2%), monthly (11.5%), and 11.5% were unsure of the periodicity. Report interpretation was considered easy by 42.3%, while 25% had never consulted one. Reasons for inconsistent dosimeter use were assessed, with forgetfulness being the most frequently reported reason (50%) (Appendix D).

When profession was examined in relation to whole-body dosimeter use, all radiographers reported using it always (76.9%) or frequently (23.1%), indicating higher compliance than that observed among nurses (54.8% always and 9.7% frequently) and physicians (62.5% always and 12.5% frequently) (Appendix E).

### 3.5. Perceived Level of Occupational Risk

**Figure 3:**
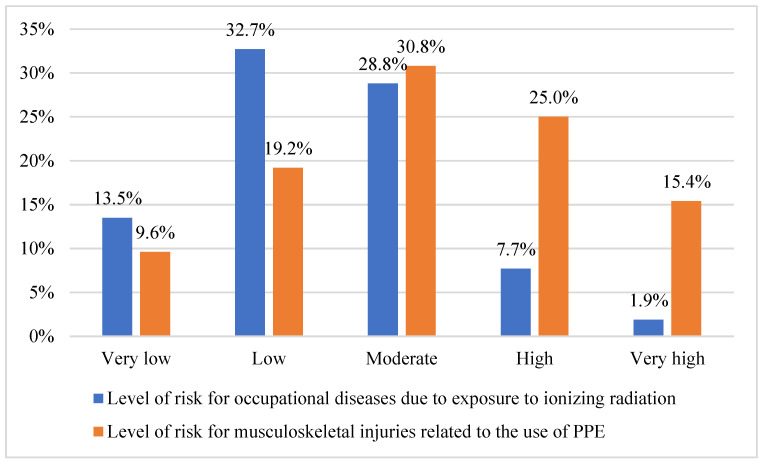
Perceived risk reported by workers (n = 52) for occupational diseases associated with ionizing radiation exposure and musculoskeletal injuries related to PPE use.

Almost half of the workers (46.2%) perceived the risk of developing radiation-related diseases as very low or low. In contrast, 40.4% of participants considered the risk of developing work-related musculoskeletal injuries to be high or very high, particularly those associated with PPE use (Figure 3).

Almost all workers (90.4%) reported never having experienced any occupational disease or work-related musculoskeletal injury associated with the use of protective equipment. In contrast, 9.6% reported experiencing some issues, although none had been formally recognized as occupational diseases. Regarding awareness of cases among colleagues, 86.5% reported not knowing anyone affected, while 13.5% reported knowing workers who had developed conditions related to such exposures.

### 3.6. Associations with Protective Equipment Use

No significant associations were found between sex and appropriate use of the apron/skirt-coat (*p* = 1.000; aOR = 0.66) or thyroid shield (*p* = 0.723; aOR = 0.43). Age ≤ 49 years was associated with appropriate use of both pieces of equipment (apron/skirt-coat: *p* = 0.020; aOR = 0.22; thyroid shield: *p* = 0.038; aOR = 0.23). Weekly exposure of 6–18 h increased the likelihood of inappropriate use (apron/skirt-coat: *p* = 0.016; aOR = 4.74; thyroid shield: *p* = 0.048; aOR = 3.02).

Cumulative exposure time showed no significant associations (*p* > 0.05; aOR = 0.71 and 1.75). RP training was positively associated with appropriate use of both protective equipment (apron/skirt-coat: *p* = 0.001; aOR = 9.36; thyroid shield: *p* = 0.020; aOR = 4.04). Perceived occupational disease risk showed a significant association only with thyroid shield use (*p* = 0.010), whereas perceived musculoskeletal risk was not significantly associated with either equipment (*p* > 0.05) (Table 2).

### 3.7. Associations with Monitoring Device Use

No statistically significant associations were observed for sex, age, weekly exposure hours, cumulative exposure time, perceived risk of occupational diseases, or perceived risk of musculoskeletal injuries related to the use of protective equipment (*p* > 0.05). RP training was significantly associated with the appropriate use of dosimeters (χ^2^(1) = 8.125; *p* = 0.004). In the unadjusted analysis, trained professionals had higher odds of using an appropriate dosimeter (OR = 5.73; 95% CI:1.65–19.94), and this association remained statistically significant after adjustment for age and sex (aOR = 5.51; 95%CI: 1.57–19.34) (Table 3).

### 3.8. Associations Between Radiation Protection Training and Perceived Occupational Risk

A high-risk perception was more prevalent among trained professionals (30.8%) than among those without training (11.5%); however, this association did not reach statistical significance (χ^2^(1) = 2.88, *p* = 0.090; aOR = 3.44). Similarly, no statistically significant differences were observed between groups regarding perceived musculoskeletal injury risk associated with protective equipment use (χ^2^(1) = 2.00; *p* = 0.158; aOR = 2.42) (Table 4).

### 3.9. Differences Among Professional Groups in Personal Protective Equipment and Dosimeter Use, and Risk Perception

Radiographers presented the highest median level of appropriate use of the apron/skirt-coat (Me = 5.0; IQR = 5.0–5.0), followed by nurses (Me = 5.0; IQR = 4.0–5.0) and physicians (Me = 4.5; IQR = 1.0–5.0), with statistically significant differences between professional groups (*p* = 0.013). Radiographers also showed the highest mean rank (35.00), followed by nurses (24.61) and physicians (20.00). Post hoc comparisons showed significantly higher use among radiographers compared with physicians (adjusted *p* = 0.024) and nurses (adjusted *p* = 0.038), with no significant difference between physicians and nurses (adjusted *p* = 1.000).

Similarly, for the thyroid shield, radiographers again demonstrated the highest median of appropriate use (Me = 5.0; IQR = 5.0–5.0), followed by nurses (Me = 5.0; IQR = 4.0–5.0) and physicians (Me = 4.0; IQR = 1.0–5.0), with statistically significant differences between groups (*p* = 0.032). Radiographers presented the highest mean rank (33.00), followed by nurses (25.95) and physicians (18.06). Post hoc analyses indicated a significant difference between radiographers and physicians (adjusted *p* = 0.029), whereas no significant differences were observed for the remaining pairwise comparisons.

**Figure 4:**
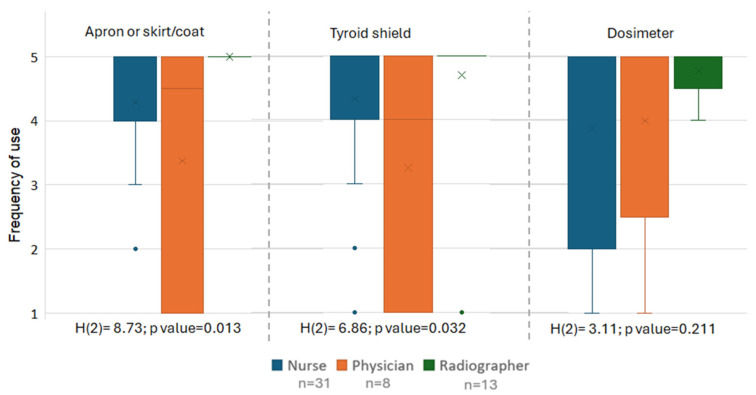
Frequency of use of the apron/skirt-coat, thyroid shield, and dosimeter across professional groups. Note: horizontal line = median; box = interquartile range (Q1–Q3); whiskers = range excluding outliers; x = mean; points = outliers.

Radiographers presented the highest median level of appropriate dosimeter use (Me = 5.0; IQR = 4.5–5.0), followed by nurses (Me = 5.0; IQR = 2.0–5.0) and physicians (Me = 5.0; IQR = 2.5–5). However, no statistically significant differences were found between professional groups (*p* = 0.211), although radiographers had the highest mean rank (32.00), followed by physicians (26.06) and nurses (24.31) (Figure 4).

No statistically significant differences were observed among professional groups regarding the perceived level of risk for occupational diseases associated with exposure to ionizing radiation (*p* = 0.606).

**Figure 5:**
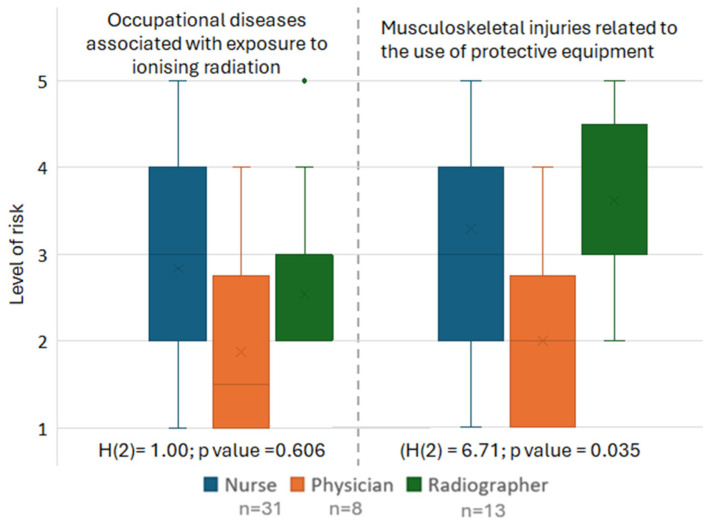
Perceived risk of radiation-related occupational diseases and musculoskeletal injuries, by professional group. Note: horizontal line = median; box = interquartile range (Q1–Q3); whiskers = range excluding outliers; x = mean; points = outliers.

Regarding the perceived risk of musculoskeletal injuries associated with the use of protective equipment, radiographers reported higher median risk levels (Me = 3.0; IQR = 3.0–4.5) than nurses (Me = 3.0; IQR = 2.0–4.0) and physicians (Me = 2.0; IQR = 1.0–2.75), with statistically significant differences between groups (*p* = 0.035). Radiographers also had the highest mean rank (31.23), followed by nurses (27.58) and physicians (14.63). Post hoc comparisons revealed that radiographers perceived a significantly higher risk than physicians (*p* = 0.036), whereas no significant differences were observed between physicians and nurses (*p* = 0.079) or between nurses and radiographers (*p* = 1.000) (Figure 5).

## 4. Discussion

In this study, RP practices and dosimeter use were assessed among healthcare professionals involved in fluoroscopy-guided procedures. The results indicate suboptimal compliance and suggest that training and occupational factors may influence protective behaviors.

About half of the participants reported having received RP training, with higher coverage among radiographers, reflecting their academic background [13,14]. Most professionals reported that their last training was more than 4 years ago. RP training was associated with higher compliance with protective equipment and dosimeter use; however, training alone is unlikely to be sufficient without institutional support, monitoring, and adequate protective resources [15].

Compliance with mandatory body-worn protective equipment remained suboptimal but within internationally reported ranges [7]. Barriers such as weight, discomfort, and hygiene concerns are well-documented and continue to limit consistent use [6,7,8,13,16]. The reference for skirt–coat configurations likely reflects improved weight distribution, supporting the need for lighter, ergonomically adapted equipment and the individualized assignment of protective devices [17].

Use of protective devices, including lead glasses and mobile, table- and ceiling-mounted shields, was limited and largely attributable to unavailability rather than noncompliance. This underscores the influence of organizational factors on RP practices. Insufficient provision and maintenance of collective shielding, particularly in operating theaters and gastroenterology units, may increase occupational exposure and should be addressed at the institutional level [18,19,20].

Compliance with whole-body dosimeter use was inconsistent, with about one-quarter of workers reporting inadequate use, potentially leading to underestimation of exposure [21]. These results align with previously documented ranges (5–98%), with forgetfulness being the predominant reason [13,22,23]. Radiographers showed higher compliance with dosimeter use than nurses and physicians, consistent with previous studies [14]; However, this difference was not statistically significant, likely due to the limited sample size, particularly among physicians (n = 8), which reduced the statistical power to detect real differences [24]. Dosimeter use is influenced by operational barriers and limited risk perception [15]. These findings support organizational strategies to promote dosimeter use across professional groups.

In Portugal, a whole-body dosimeter is typically used at trunk level (chest or abdomen) to assess individual dose equivalent, measuring Hp(10) and Hp(0.07). The use of additional dosimeters, such as extremity dosimeters, is recommended only when higher exposure is expected, based on risk assessment, rather than being routinely required. The extremity dosimeter (ring/bracelet) is additional, used if the dose to the extremities exceeds 30% of the legal dose limit, and also measures Hp(0.07) and is placed near the extremity closest to the source [2]. The eye lens dosimeter, placed near the eye closest to the source, measures Hp(3) and is the most accurate [2,25,26].

When the dosimeter is placed at chest level, the dose may be overestimated or underestimated depending on whether it is placed over or under the PPE [3,26]. The ICRP recommends using one dosimeter inside the PPE and another outside at collar level for a more accurate estimate of the effective dose and the dose to the eye lens [3,25,27]. However, this practice was not observed in any participant in this study.

Age, but not sex, was associated with PPE compliance, with professionals aged 50 years or older showing higher odds of inadequate use of the apron/skirt–coat and thyroid shield. Higher weekly exposure load was also linked to poorer compliance, whereas cumulative years of work were not. Several authors have reported musculoskeletal problems associated with the use of protective equipment [28,29,30], suggesting that physical limitations associated with aging, discomfort, or fatigue from prolonged use may compromise compliance with protective measures. These results support targeted training and interventions for older professionals and those with higher workloads.

Nearly half of the participants perceived their risk of radiation-related disease as low or very low, a lower risk perception than that reported by Chow et al. [31]. This may reflect underestimation of risk or confidence in protective equipment. Although risk perception was not statistically associated with dosimeter or apron/skirt–coat use, descriptive trends suggested higher compliance among professionals with greater perceived risk.

Risk perception is subjective and varies according to each professional’s experience, knowledge of the effects of radiation, and their perception of immediate versus long-term risks. While the risk of radiation-related diseases is real, the fact that many participants rated the risk as low may reflect a more limited understanding of less immediate diseases, such as cancer, which are harder to directly associate with daily exposure. In contrast, conditions like cataracts, which are more tangible, may lead to a higher risk perception, as observed in Chow et al.’s study [31]. Therefore, the low risk perception, while valid in the context of the study, does not necessarily reflect the actual risk of radiation exposure.

The association between training and higher risk perception suggests that trained workers are more aware of occupational risks. Although causality cannot be inferred, the findings indicate a potentially bidirectional relationship among risk perception, training, and compliance with protective equipment.

Radiographers showed higher PPE compliance than other professional groups, suggesting that risk perception may influence safety behaviors but is unlikely to be the primary driver of protective equipment use.

No statistically significant differences in risk perception were observed between professional groups, despite slight numerical variations. This partially contrasts with findings by Pinela et al. [32] and may reflect limited statistical power due to sample size [24], particularly after Bonferroni correction [33]. Alternatively, homogeneous risk perception across groups may be influenced by accumulated clinical experience, risk normalization, or standardized institutional practices.

Ideally, the perception of risk related to radiation-related diseases should be balanced. The perception should be high enough to encourage professionals to adopt the necessary precautions and become aware of the real risks of radiation, but not so high as to generate excessive anxiety or fear. Continuous radiation protection training is essential to achieving this balance. Education on the long-term effects of radiation, such as cancer, and the effectiveness of PPE can help increase risk perception in a controlled manner, without inducing panic [13].

Ergonomic risk perception differed significantly across professions, with radiographers reporting higher perceived risk than physicians. Longer exposure to fluoroscopic procedures among radiographers may partly explain this difference. Physicians may underestimate ergonomic risks due to lower exposure to equipment weight and prioritization of procedural performance, a behavior also described in the literature [19]. Similar perceptions between radiographers and nurses may reflect comparable physical demands. Overall, ergonomic risks appear to be more distinctly perceived across professional groups than radiation-related risks, possibly because musculoskeletal effects are immediate and tangible, whereas radiation risks are cumulative and less perceptible.

As this is a case study conducted in a single healthcare unit, the findings reflect a local context and may not be generalizable. The relatively low response rate, although comparable to that reported in studies of workers exposed to ionizing radiation [20,34,35,36], together with the small sample size, may have limited statistical power and generalizability.

The use of a self-administered questionnaire entails inherent limitations, including potential social desirability bias, recall bias, and subjective interpretation of the questions. Social desirability bias may lead participants to adjust their responses to what they perceive as socially acceptable, particularly in topics such as the use of PPE and dosimeters in RP. Recall bias can affect response accuracy, especially when participants are asked to report past exposures or repetitive behaviors. Subjective interpretations of the questions may also vary among participants, influencing how they are understood and answered.

In addition, voluntary participation may introduce selection bias, and the response rate raises the possibility of non-response bias, as individuals with greater interest or awareness of RP may have been more likely to participate.

The small number of physicians limits the robustness of subgroup analyses; therefore, these findings should be interpreted with caution and considered exploratory.

Despite these limitations, common to cross-sectional observational studies, several measures were implemented to mitigate bias, including anonymous data collection, the use of a structured instrument, and pre-testing of the questionnaire.

Future studies should combine self-reported data with objective measurements, audits, or electronic monitoring and include multicenter designs with larger samples to better characterize organizational and behavioral determinants of RP compliance.

## 5. Conclusions

Inadequate use of protective equipment and dosimeters may lead to unnecessary exposure and the underestimation of occupational radiation doses, increasing workers’ vulnerability to the cumulative effects of radiation. The findings indicate that RP training is a key determinant of compliance and proper use of protective equipment and individual dosimeters. Consequently, it is crucial to reinforce and regularly update such training, promoting correct use of protective equipment and monitoring devices while aligning risk perception with the potential long-term effects of occupational exposure. The technical knowledge and safety culture promoted through training serve as critical factors in mitigating radiation-related risks.

At the institutional level, the findings emphasize the need for hospital management to prioritize the procurement of ergonomic, lightweight protective equipment to mitigate physical fatigue, which is a key barrier to compliance. Additionally, PPE should be tailored to individual body types to improve comfort and reduce the risk of musculoskeletal injuries. Furthermore, prioritizing collective protective equipment is crucial to ensuring comprehensive protection for all workers, potentially avoiding the need for PPE or adding protection to minimize the exposure dose.

The institution should also implement targeted RP programs that extend beyond theoretical knowledge, focusing on practical, department-specific training. These programs should incorporate task rotation, ergonomic positioning during procedures, and the promotion of stretching exercises between procedures to enhance workers’ physical well-being.

Furthermore, fostering a proactive safety culture within the institution is crucial. This culture should actively monitor and support the consistent use of protective equipment and personal dosimeters, ensuring that RP practices are maintained continuously and effectively, thereby promoting a safer and healthier work environment.

## Figures and Tables

**Table 1:** Sociodemographic characterization of the sample and professional activity (n = 52).

Variables		n	%
Sex	Female	42	80.8
	Male	10	19.2
Age group	≤29 years	2	3.8
	30–39 years	13	25.0
	40–49 years	18	34.6
	50–59 years	17	32.7
	≥60 years	2	3.8
Academic degree	Bachelor’s degree	36	69.2
	Master’s degree	14	26.9
	Doctoral degree	2	3.8
Profession	Nurse	31	59.6
	Physician	8	15.4
	Radiographer	13	25.0
Exposure context	Operating room	23	44.2
	Interventional procedures	16	30.8
	Operating room and interventional procedures	8	15.4
	Gastroenterology	5	9.6
Exposure time (years)	≤1 year	2	3.8
	2–10 years	18	34.6
	11–20 years	13	25.0
	21–30 years	11	21.2
	≥31 years	8	15.4
Number of employers (n)	1	49	94.2
	2	2	3.8
	≥3	1	1.9
Exposure time (hours/week)	≤6 h	28	53.8
	7–12 h	15	28.8
	13–18 h	5	9.6
	19–24 h	1	1.9
	25–30 h	1	1.9
	31–36 h	2	3.8
Radiation protection training	0	26	50
	1	10	19.2
	2–5	12	23.1
	≥6	4	7.7
Time since last radiation protection training (years) (n = 26)	≤1 year	4	7.7
	2–3 years	3	5.8
	≥4 years	19	36.5

n = absolute frequency; % = relative frequency.

**Table 2:** Associations with equipment protective use.

			Use of the Apron or Skirt/Coat		*p*-Value	Odds Ratio [95% CI]	Use of the Thyroid Shield		*p*-Value	Odds Ratio [95% CI]
			Inappropriate (n = 17)	Appropriate (n = 35)			Inappropriate (n = 18)	Appropriate (n = 34)		
Sex	Female	n	14	28	1.000	1.17 [0.26, 5.21]	14	28	0.723	0.75 [0.18, 3.10]
		(%)	(82.4)	(66.7)			(77.8)	(82.4)		
	Male	n	3	7		0.66 [0.13, 3.45] ^†^	4	6		0.43 [0.09, 2.06] ^†^
		(%)	(17.6)	(70.0)			(22.2)	(17.6)		
Age (years)	≤49	n	7	26	0.020 *	0.24 [0.07, 0.83] *	8	25	0.038 *	0.29 [0.09, 0.96] *
		(%)	(41.2)	(74.3)			(44.4)	(73.5)		
	≥50	n	10	9		0.22 [0.06, 0.82] ^‡^*	10	9		0.23 [0.06, 0.85] ^‡^*
		(%)	(58,8)	(25.7)			(55.6)	(26.5)		
Hours/week (hours)	≤6–18	n	15	19	0.016 *	6.32 [1.25, 31.86] *	15	19	0.048 *	3.95 [0.96, 16.21]
		(%)	(88.2)	(54.3)			(83.3)	(55.9)		
	19–36	n	2	16		4.74 [0.89, 25.17] ^†‡^	3	15		3.02 [0.69,13.30] ^†‡^
		(%)	(11.8)	(45.7)			(16.7)	(44.1)		
Cumulative exposure time (years)	≤20	n	4	16	0.123	0.37 [0.10; 1.34]	6	14	0.580	0.71 [0.22; 2.36]
		(%)	(23.5)	(45.7)			(33.3)	(41.2)		
	≥21	n	13	19		0.71 [0.15, 3.37] ^†‡^	12	20		1.75 [0.36, 8.48] ^†‡^
		(%)	(76.5)	(54.3)			(66.7)	(58.8)		
Radiation protection training	No	n	14	12	0.001 *	8.94 [2.14, 37.34] *	13	13	0.020 *	4.20 [1.21, 14.54] *
		(%)	(82.4)	(34.3)			(72.2)	(38.2)		
	Yes	n	3	23		9.36 [2.06, 42.52] ^†‡^*	5	21		4.04 [1.10, 14.82] ^†‡^*
		(%)	(17.6)	(65.7)			(27.8)	(61.8)		
Perceived occupational disease risk	Low	n	16	25	0.078	6.40 [0.75, 54.90]	18	23	0.010 *	ND
		(%)	(94.1)	(71.4)			(100)	(67.6)		
	High	n	1	10		6.15 [0.66, 57.43] ^†‡^	0	11		
		(%)	(5.9)	(28.6)			(0)	(32.4)		
Perceived musculoskeletal injury risk (related to protective equipment)	Low	n	13	18	0.084	3.07 [0.84, 11.29]	14	17	0.052	3.50 [0.96, 12.83]
		(%)	(76.5)	(51.4)			(77.8)	(50.0)		
	High	n	4	17		2.58 [0.59, 11.39] ^†‡^	4	17		2.62 [0.60, 11.53] ^†‡^
		(%)	(23.5)	(48.6)			(22.2)	(50.0)		

Notes: Appropriate use = “always”; Inappropriate use = “never”, “rarely”, “sometimes”, or “frequently”; Level risk low = “very low”, “low”, “moderate”; Level risk high = “high” and “very high”; Fisher’s was used for sex and occupational disease risk, whereas χ^2^ tests were applied to all remaining variables. * *p*-value < 0.05—significant difference; ^†^ adjusted for age ^‡^ adjusted for sex; ND = not defined.

**Table 3:** Associations with monitoring device use.

			Dosimeter Use		*p*-Value	Odds Ratio [95% CI]
			Inappropriate (n = 20)	Appropriate (n = 32)		
Sex	Female	n (%)	16 (80.0)	26 (81.3)	1.000	0.92 [0.23, 3.78] 0.74 [0.17, 3.25] ^†^
	Male	n (%)	4 (20.0)	6 (18.8)		
Age (years)	≤49	n (%)	11 (55.0)	22 (68.8)	0.316	0.56 [0.18, 1.76] 0.52 [0.15, 1.74] ^‡^
	≥50	n (%)	9 (45.0)	10 (31.3)		
Hours/week (hours)	≤6–18	n (%)	16 (80.0)	18 (56.3)	0.080	3.11 [0.85, 5.39] 2.83 [0.73, 10.89] ^†‡^
	19–36	n (%)	4 (20.0)	14 (43.8)		
Cumulative exposure time (years)	≤20	n (%)	5 (25.0)	15 (46.9)	0.115	0.38 [0.11, 1.29] 0.41 [1.00, 1.67] ^†‡^*
	≥21	n (%)	15 (75.0)	17 (53.1)		
Radiation protection training	No	n (%)	15 (57.7)	11(42.3)	0.004 *	5.73 [1.65, 19.94] * 5.51 [1.57, 19.34] ^†‡^*
	Yes	n (%)	5 (19.2)	21 (80.8)		
Perceived occupational disease risk	Low	n (%)	16 (80.0)	25 (78.1)	1.000	1.12 [0.28, 4.45] 3.18 [0.78, 12.93] ^†‡^
	High	n (%)	4 (20.0)	7 (21.2)		
Perceived musculoskeletal injury risk (related to protective equipment)	Low	n (%)	15 (75.0)	16 (50.0)	0.074	3.00 [0.88, 10.23] 3.18 [0.78, 12.93] ^†‡^
	High	n (%)	5 (25.0)	16 (50.0)		

Notes: Appropriate use = “always”; Inappropriate use = “never”, “rarely”, “sometimes”, or “frequently”; Level risk low = “very low”, “low”, “moderate”; Level risk high = “high” and “very high”; Fisher’s was used for sex and occupational disease risk, whereas χ^2^ tests were applied to all remaining variables. * *p*-value < 0.05—significant difference; ^†^ adjusted for age ^‡^ adjusted for sex.

**Table 4:** Association between radiation protection training and perceived occupational and musculoskeletal risk.

			Radiation Protection Training		*p*-Value	Odds Ratio [95% CI]
			Yes (n = 26)	No (n = 26)		
Perceived occupational disease risk	Low	n (%)	18 (69.2)	23 (88.5)	0.090	3.41 [0.79, 14.72] 3.44 [0.75, 15.80] ^†^
	High	n (%)	8 (30.8)	3 (11.5)		
Perceived musculoskeletal injury risk (related to protective equipment)	Low	n (%)	13 (50.0)	18 (69.2)	0.158	2.25 [0.72, 6.99] 2.42 [0.64, 9.15] ^†^
	High	n (%)	13 (50.0)	8 (30.8)		

Notes: Appropriate use = “always”; Inappropriate use = “never”, “rarely”, “sometimes”, or “frequently”; High risk = “very high”, “high”; Low risk = “very low”, “low”, “moderate”. Statistical tests—χ^2^; * *p*-value < 0.05—significant difference; ^†^ adjusted for age and sex.

## Data Availability

The data presented in this study are available on request from the corresponding author. Restrictions apply to the datasets. The data are not publicly available due to a large part of the article’s data is part of an ongoing PhD study, so the raw data cannot be freely accessible at this stage, and also due to data protection concerns.

## Abbreviations

aOR—adjusted odds ratios; CI—confidence intervals; IQR—interquartile ranges; OR—odds ratios; PPE—personal protective equipment; RP—radiation protection.

## Appendix A

Distribution of radiation protection training (n = 26): type of training (A) and distribution by professional group (B).

## Appendix B

Availability of protective equipment at the institution reported by the workers (n = 52): personal protective equipment and collective protective equipment available (A) and personal protective equipment for exclusive use (B).

## Appendix C

Reasons for not using personal protective equipment and collective protective equipment.

**Reasons for Not Using Personal or Collective Equipment**	**Lead Glasses**		**Lead Aprons/Skirt-Coat**		**Thyroid Shields**		**Lead Gloves**		**Mobile Shields**		**Suspended Shields**		**Table Skirt Shields**		**Protective Drapes**	
	**n**	**%**	**n**	**%**	**n**	**%**	**n**	**%**	**n**	**%**	**n**	**%**	**n**	**%**	**n**	**%**
Non-existence	16	21.6%	1	1.3%	2	3.3%	23	37.1%	36	67.9%	27	50.0%	24	43.6%	39	73.6%
Weight	2	2.7%	11	13.8%	2	3.3%										
Pain			5	6.3%												
Inadequate disinfection	1	1.4%	3	3.8%	4	6.7%										
Lack of information	1	1.4%														
Difficult access	1	1.4%					1	1.6%								
Unavailability	8	10.8%	2	2.5%	2	3.3%	3	4.8%								
Discomfort	7	9.5%	7	8.8%			1	1.6%								
Forgetfulness	7	9.5%	2	2.5%	2	3.3%					3	5.6%	4	7.3%		
I don’t need it because I am far enough away	6	8.1%	7	8.8%			9	14.5%			3	5.6%	2	3.6%	2	3.8%
Visual acuity	7	9.5%														
Compatibility with prescription glasses	4	5.4%														
Not having one’s own equipment	8	10.8%			1	1.7%										
Does not apply to the work area	4	5.4%	3	3.8%	3	5.0%			10	18.9%	13	24.1%	13	23.6%	10	18.9%
Lack of training/information							1	1.6%					1	1.8%		
Dirtiness			1	1.3%	5	8.3%										
Limits movement			3	3.8%												
Inadequate storage			2	2.5%	4	6.7%										
Heat			1	1.3%	2	3.3%										
Inadequate size			2	2.5%			5	8.1%								
I don’t need it because I have mobile shielding			1	1.3%	1	1.7%	1	1.6%								
Does not apply because I always use it	2	2.7%	29	36.3%	31	51.7%	2	3.2%	4	7.5%	3	5.6%	11	20,0%		
Loss of sensitivity							6	9.7%								
Limits movement							5	8.1%								
Incompatible with the work									2	3.8%	4	7.4%			2	3.8%
I don’t need it because I have PPE									1	1.9%	1	1.9%				
Other			1 *			1.7%										
Total		100%		100%		100%		100%		100%		100%		100%		100%
Note: * thyroidectomized.																

## Appendix D

Data related to individual monitoring.

**Question**	**Response**	**n**	**%**	
Does the employer provide individual monitoring using a dosimeter?	Yes—public sector	52	100.0	
Type of dosimeter used	− 1 whole-body dosimeter	35	67.3	
	− 1 whole-body dosimeter and 1 ring dosimeter	15	28.9	
	− 2 whole-body dosimeters	1	1.9	
	− 2 whole-body dosimeters and 1 ring dosimeter	1	1.9	
Location of the individual dosimeter when only one whole-body dosimeter is used	− At chest level underneath the apron or vest (or collar)	43	82.7	
	− At chest level underneath the apron or vest, and at chest level on top of the apron or vest	1	1.9	
	− At chest level on top of the apron or vest	5	9.6	
	− In the pocket of the apron, vest, or skirt	2	3.8	
	− Underneath the thyroid collar/shield	1	1.9	
Do you have access to the dosimeter reading report?	− No—public sector	12	23.1	
	− Yes—public sector	40	76.9	
□ Evaluation of the dosimeter	− Monthly	10	19.2	
	− Quarterly	24	46.2	
	− I don’t know	6	11.5	
□ Easy understanding the dosimeter report	− Yes	22	42.3	
	− No	5	9.6	
	− I don’t know/I have never seen any	13	25.0	
□ Access to accumulated values if you work for more than one employer	− No	3	5.8	
	− I do not work for more than one employer	36	69.2	
	− Yes	1	1.9	
Reasons for not using the individual dosimeter always	− Forgetfulness	26	50.0	
	− Does not apply because I always use it	22	42.3	
	− Lack of information or training	2	3.8	
	− Fear of losing it	2	3.8	
	− Use is unnecessary	1	1.9	
	− Discomfort	1	1.9	
	− Other	2	3.8	

## Appendix E

Profession and frequency of whole-body dosimeter use (n = 52).
